# The Impact Of Exercise On Inflammation, Oxidative Stress And Remodelling In Cardiac Muscle

**DOI:** 10.36660/abc.20190735

**Published:** 2020-01

**Authors:** Fernando Mendes, Diana Martins

**Affiliations:** 1 Politécnico de Coimbra - Departamento de Ciências Biomédicas Laboratoriais ESTeSC, Coimbra - Portugal; 2 Consórcio CNC.IBILI/Centro de Biomedicina Inovadora e Biotecnologia (CIBB), Universidade de Coimbra, Coimbra - Portugal; 3 Instituto de Biofísica e Instituto de Investigação Clínica e Biomédica de Coimbra (iCBR) área de Meio Ambiente Genética e Oncobiologia (CIMAGO), Faculdade de Medicina, Universidade de Coimbra, Coimbra - Portugal; 4 European Association for Professions in Biomedical Sciences, Bruxelles - Belgium; 5 I3S, Instituto de Investigação e Inovação em Saúde, Universidade do Porto, Porto - Portugal

**Keywords:** Coronary Artery Disease, Exercise, Menopause, Dyslipidemias, Physical activity, Collagen, Oxidative Stress, Inflammation, Mice

Aging is naturally connected with a decline, in some if not all physiological functions, namely loss of bone mass density (BMD) and strength associated with the loss of muscle mass.^1-3^

This muscle loss happens mainly due to an imbalance between muscle protein synthesis and muscle protein breakdown. The increase of catabolic factors such as oxidative stress and inflammation contribute significantly to the above process. Nevertheless, the weakening of strength and muscle loss is not linear, occurring differently in all sexes. Other factors such as a decline in hormonal levels due to menopause phenomenon are thought to be also implicated in this process.^1^ In fact, some authors proposed that, in females, an accelerated loss of muscle mass and strength arises at an earlier age than in males, around the time of menopause.^1^ A good body of evidence supports that the decline in muscle mass may be in line with the estrogen decrease that typifies menopausal years.^3^

The present study published at *Arquivos Brasileiros de Cardiologia* by Brianezi et al.^4^ proposed to investigate the aerobic exercise training on the left ventricle in low-density lipoprotein (LDL) knockout ovariectomized mice, mimicking the effects of menopause, exercise and its effects on muscles of the mouse.

It is well known that estrogen decrease contributes to the loss of BMD, as well as to the redistribution of subcutaneous fat to the visceral area, associated to an increased risk of cardiovascular disease, affecting the quality of life of females. To worsen the undesirable impact of menopause on female’s health, the decrease in estrogen levels may also have a direct effect on muscle tissue.^1,3^

In order to better understand the above-mentioned effects, Ledimar et al.,^4^ design the following experiment; a group of thirty animals were divided into 6 groups, each with 5 mice: non-ovariectomized sedentary control, ovariectomized sedentary control, ovariectomized trained control, non-ovariectomized sedentary LDL knockout, ovariectomized sedentary LDL knockout, ovariectomized LDL knockout trained. The animals were ovariectomized at 9 months according to the protocol described, after that the animals were exposed to two different tests, one regarding the max training test and a second one training exercises. Then animals were euthanized and parameters such as the average volume density type I and III collagen fibres, matrix metalloproteinases (MMP) 2 and 9, oxidative stress (OS) were analysed. Their results revealed that the exercise protocol altered the volume of collagen fibres in type I and collagen remodelling parameters namely MMP-2, and further reduced the OS parameter of 8-hydroxy-2’-deoxyguanosine (8-OhdG).

In females, aging of the vascular system only occurs due to a decline of ovarian function along with the decline in associated circulating hormones, in particular estrogen.^5^ Although the risk factors, age and estrogen deficiency are well characterized, the mechanism of estrogen action in the vasculature compromised by aging are not well determined. Inflammation is associated with the aging of the vascular system, mainly due to the elevated levels of proinflammatory cytokines, such as tumour necrosis factor (TNF), which is also known to induce MMP. For example, MMP2 can specifically cleavage big endothelin-1, suggesting the role of this proteolytic enzyme in the vascular wall. Also, an interesting study found increased MMP2 activity in the mesenteric arteries from aged/estrogen-deficient animals, which was restored when the animals were treated with an anti-inflammatory agent targeting TNF.^6^ Also, the major source of MMP-9 are leucocytes, major key players in inflammatory process, suggesting that MMPs are critical modulators of vascular disease in an aging/estrogen-deficient model.

Although estrogen replacement had been proposed for the management of cardiovascular risk associated with aging in female, its efficacy is controversial. Physical activity seems to be an effective alternative to estrogen supplementation in post-menopausal females, improving aerobic fitness and physiological adaptations of the cardiovascular system.^7-9^

There is some intriguing evidence related to the loss of estrogen in mice showing a role in muscle contractile properties. Wohlers et al.^10^ studied the contractile properties of ovariectomized mice muscle, has demonstrated the lower capability of activating adenosine monophosphate kinase (AMPK) phosphorylation.^10^ This protein is important for glucose uptake and lipid oxidation in muscle, being implicated in energy production, needed to produce muscle contractions.^1,11,12^

Associated to physical activity is the generation of reactive species of oxygen (ROS) that may damage cell membranes lipids, proteins as well as both mitochondrial and nuclear DNA in case of oxidative stress, that can result in serious or mortal cellular injury.^13,14^

Novais et al.^15^ reported that with eight weeks of exercise training in the trained hypertensive menopausal group, aerobic training was effective in promoting an increase in superoxide dismútase (SOD) and catalase, antioxidant agents, which have a crucial role in oxidative stress modulation. Also in animal studies, Claudio et al.^16^ demonstrated that eight weeks of interval training programs significantly increased SOD and catalase expression, contributing to the reduction of cardiac superoxide production in rats with ovariectomized, in this case with hypertension, which may prevent coronary heart disease in hypertensive postmenopausal women.^1,7,14^

Brianezi et al.^4^ conclude that moderate-intensity aerobic training acts on the volume of collagen fibres and on the collagen remodelling, with reduced oxidative stress in left ventricles of mice ovariectomized LDLr Knockout. It is known that after menopause, different factors contribute to the decline in muscle mass, the combination of physical inactivity, protein intake and oxidative stress^16^ clearly contribute the increase to this process.^16^ Associated with the oxidative stress increase, characterized by the imbalance between the production and the removal of free radicals, usually due to an inadequate antioxidant system.^16,17^ Oxidative stress is related to a higher ROS production from the mitochondria, which can induce cell apoptosis.^18^ Meaning that the mitochondrial DNA may be damaged due to oxidative stress, affecting mitochondria capacity to produce energy, contributing to a higher susceptibility to apoptosis which finally intensifies muscle fibre atrophy or death and muscle mass decrease.^1^

Taking together, the results of Brianeziet et al.^4^ conclude that physical exercise contributes positively influencing the control and dyslipidemic groups in the parameter of density and volume of collagen fibres of type I and the control group in relation to oxidative stress. The mechanisms underlying the pathophysiology of menopausal alterations are complex and implicate estrogen-MMP-metabolic deregulation with age-driven vascular changes.
